# Association of Bilateral Brachial-Ankle Pulse Wave Velocity Difference with Peripheral Vascular Disease and Left Ventricular Mass Index

**DOI:** 10.1371/journal.pone.0088331

**Published:** 2014-02-13

**Authors:** Ho-Ming Su, Tsung-Hsien Lin, Po-Chao Hsu, Wen-Hsien Lee, Chun-Yuan Chu, Szu-Chia Chen, Chee-Siong Lee, Wen-Chol Voon, Wen-Ter Lai, Sheng-Hsiung Sheu

**Affiliations:** 1 Division of Cardiology, Department of Internal Medicine, Kaohsiung Medical University Hospital, Kaohsiung Medical University, Kaohsiung, Taiwan; 2 Department of Internal Medicine, Kaohsiung Municipal Hsiao-Kang Hospital, Kaohsiung Medical University, Kaohsiung, Taiwan; 3 Faculty of Medicine, College of Medicine, Kaohsiung Medical University, Kaohsiung, Taiwan; 4 Division of Nephrology, Department of Internal Medicine, Kaohsiung Medical University Hospital, Kaohsiung Medical University, Kaohsiung, Taiwan; University of Milan, Italy

## Abstract

Unequal arterial stiffness had been associated with cardiovascular risks. We investigated whether an association existed between unequal arterial stiffness indicated by bilateral brachial-ankle pulse wave velocity (baPWV) difference and ankle-brachial index (ABI), baPWV, echocardiographic parameters and interarm and interankle systolic blood pressure (BP) differences. A total of 1111 patients referred for echocardiographic examination were included in this study. The BPs, ABI and baPWV were measured simultaneously by an ABI-form device. The ΔbaPWV was defined as absolute value of difference between bilateral baPWV. We performed three multivariate analyses for determining the factors associated with a ΔbaPWV ≧ 185 cm/s (90 percentile of ΔbaPWV) (model 1: significant variables in univariate analysis and ABI <0.9 and baPWV; model 2: significant variables in univariate analysis and left ventricular mass index [LVMI]; model 3: significant variables in univariate analysis and interankle systolic BP difference ≧ 15 mmHg). The ABI <0.9 and high baPWV (both *P*<0.001) in model 1, high LVMI (*P* = 0.021) in model 2 and an interankle systolic BP difference ≧ 15 mmHg (*P* = 0.026) in model 3 were associated with a ΔbaPWV ≧ 185 cm/s, but the interarm systolic BP difference ≧ 10 mmHg was not (*P* = NS). Our study demonstrated ABI <0.9, high baPWV, high LVMI and an interankle systolic BP difference ≧ 15 mmHg were associated with unequal arterial stiffness.

## Introduction

Arterial stiffness is an independent predictor for adverse cardiovascular outcomes in various populations [1]–[3]. Although arterial stiffness exhibits systemic change, there is often unequal progression in different artery territories [4]–[6]. A recent study demonstrated that arterial stiffness in different regions might have different roles in cardiovascular disease, and showed that arterial stiffening did not occur equally among the arterial regions in patients with ischemic heart disease [4]. However, the exact mechanisms responsible for the correlation between unequal arterial stiffness and adverse cardiovascular outcomes remain unclear.

Previous studies have found there was a significant association of a blood pressure (BP) difference between arms and ankles with peripheral vascular disease, pre-existing coronary artery disease, left ventricular hypertrophy (LVH) and increased cardiovascular mortality and all-cause mortality [7]–[13]. The large interarm or interankle BP difference might result from unequal limb atherosclerosis and then be associated with peripheral vascular disease, LVH and poor cardiovascular outcomes. The hypothesis that an association exists between an interarm and interankle BP difference and unequal arterial stiffness has never been examined.

Current technology has allowed for simultaneous BP measurement in 4 limbs, [14] which might provide a comprehensive evaluation of BP differences between 4 limbs, such as ankle-brachial index (ABI) and the interarm and interankle BP differences. A clinical device, ABI-form (Colin VP1000, Komaki, Japan), has been developed to automatically and simultaneously measure BPs in both arms and ankles and record pulse waves of the brachial and posterior tibial arteries using an automated oscillometric method. Using this device, we can easily obtain the values of brachial-ankle pulse wave velocity (baPWV) and ABI, good markers for arterial stiffness and peripheral artery occlusive disease respectively [14], [15].

Accordingly, the aims of this study, using a technique of simultaneous BP measurement, are to compare the ABI <0.9, baPWV, echocardiographic parameters and an interarm and interankle systolic BP difference between patients with and without unequal arterial stiffness, as indicated by bilateral baPWV difference, and to identify the independent factors associated with a large bilateral baPWV difference.

## Subjects and Methods

### Study Patients and Design

Study subjects were prospectively included from a group of patients who arranged for echocardiographic examinations at Kaohsiung Municipal Hsiao-Kang Hospital. Patients with significant aortic or mitral valve diseases, atrial fibrillation, or inadequate image visualization were excluded. We did not include all patients consecutively because ABI, baPWV and BPs must be measured within 10 min after the completion of an echocardiographic examination. Finally, 1111 patients (mean age 60.7±13.7 years, 629 males/482 females) were included.

### Ethics Statement

The study protocol was approved by the Institutional Review Board of the Kaohsiung Medical University Hospital (KMUH-IRB-20130151). Informed consents have obtained in written form from patients and all clinical investigation was conducted according to the principles expressed in the Declaration of Helsinki. The patients gave consent for the publication of the clinical details.

### Evaluation of Cardiac Structure and Function

The echocardiographic examination was performed by one experienced cardiologist with a VIVID 7 (General Electric Medical Systems, Horten, Norway), with the participant respiring quietly in the left decubitus position. The cardiologist was blinded to the other data. Two-dimensional and two-dimensionally guided M-mode images were recorded from the standardized views. The Doppler sample volume was placed at the tips of the mitral leaflets to obtain the left ventricular inflow waveforms from the apical 4-chamber view. All sample volumes were positioned with ultrasonic beam alignment to flow. Pulsed tissue Doppler imaging was obtained with the sample volume placed at the lateral corner of the mitral annulus from the apical 4-chamber view. The wall filter settings were adjusted to exclude high-frequency signals and the gain was minimized. The echocardiographic measurements included left ventricular internal diameter in diastole (LVIDd), left ventricular posterior wall thickness in diastole (LVPWTd), interventricular septal wall thickness in diastole (IVSTd), E-wave deceleration time, transmitral E wave velocity (E) and early diastolic mitral annulus velocity (Ea). Left ventricular systolic function was assessed by left ventricular ejection fraction (LVEF). Left ventricular mass was calculated using Devereux-modified method, i.e. left ventricular mass = 1.04×[(IVSTd + LVIDd + LVPWTd)^3^– LVIDd^3^] –13.6 g [16]. Left ventricular mass index (LVMI) was calculated by dividing left ventricular mass by body surface area. LVH was defined as suggested by the 2007 European Society of Hypertension/European Society of Cardiology guidelines [17]. The left atrial volume was measured by the biplane area–length method [18]. Apical 4- and 2-chamber views were obtained to determine the left atrial area and length (from the middle of the plane of the mitral annulus to the posterior wall). The maximal left atrial chamber area and length were measured before mitral valve opening, excluding the left atrial appendage and pulmonary veins. Left atrial volume index (LAVI) was calculated by dividing left atrial volume by body surface area. The raw ultrasonic data were recorded and analyzed offline by a cardiologist, blinded to the other data, using EchoPAC software (GE Medical Systems).

### Assessment of ABI, baPWV and ΔbaPWV

The values of ABI and baPWV were measured by one experienced technician with an ABI-form device (VP1000; Colin Co. Ltd., Komaki, Japan), which automatically and simultaneously measures blood pressures in both arms and ankles using an oscillometric method [14]. The ABI was calculated by the ratio of the ankle systolic BP divided by the higher systolic BP of the arms. After obtaining bilateral ABI values, the lower one was used for later analysis. For measuring baPWV, pulse waves obtained from the brachial and tibial arteries were recorded simultaneously and the transmission time, which was defined as the time interval between the initial increase in brachial and tibial waveforms, was determined. The transmission distance from the arm to each ankle was calculated according to body height. The baPWV value was automatically computed as the transmission distance divided by the transmission time. After obtaining bilateral baPWV values, the higher one was used as representative for each subject. The ABI and baPWV measurements were done once in each patient. The validation of this automatic device and its reproducibility have been previously published [14]. The ΔbaPWV was defined as absolute value of difference between bilateral baPWV.

### Assessment of BP

To prevent overestimation and observer bias, interarm and interankle difference of BPs should be assessed simultaneously at both arms with one or two automated devices [19]. In our study, the bilateral arm and ankle BP measurements were done simultaneously and automatically using the ABI-form device. The systolic and diastolic BPs were measured by an appropriate cuff and the average of systolic and diastolic BPs of bilateral arms were used for later analysis.

### Collection of Demographic, Medical and Laboratory Data

Demographic and medical data including age, gender, smoking history and comorbid conditions were obtained from medical records or interviews with patients. The body mass index was calculated as the ratio of weight in kilograms divided by square of height in meters. Laboratory data were measured from fasting blood samples using an autoanalyzer (Roche Diagnostics GmbH, D-68298 Mannheim COBAS Integra 400). Serum creatinine was measured by the compensated Jaffé (kinetic alkaline picrate) method in a Roche/Integra 400 Analyzer (Roche Diagnostics, Mannheim, Germany) using a calibrator traceable to isotope-dilution mass spectrometry. [20] The value of estimated glomerular filtration rate (eGFR) was calculated using the 4-variable equation in the Modification of Diet in Renal Disease (MDRD) study [21]. Blood samples were obtained within 1 month of enrollment.

### Reproducibility

Forty-five patients were randomly selected for evaluation of the reproducibility of right baPWV and left baPWV by two separate measurements at least 5 min apart by the same technician. Mean percent error was calculated as the absolute difference divided by the average of the two observations.

### Statistical Analysis

Statistical analysis was performed using SPSS 15.0 for windows (SPSS Inc. Chicago, USA). Data were expressed as percentages, mean ± standard deviation or median (25^th^–75^th^ percentile) for ΔbaPWV, interarm and interankle systolic BP differences and triglyceride. The differences between groups were checked by Chi-square test for categorical variables or by independent *t*-test for continuous variables. Multiple logistic regression analysis was used to identify the factors associated with a ΔbaPWV ≧ 185 cm/s (90 percentiles of ΔbaPWV). Significant variables in univariate analysis were selected as covariates for multivariate analysis. A difference was considered significant if the *P* value was less than 0.05.

## Results

The mean age of the 1111 patients was 60.7±13.7 years. The median (25^th^–75^th^ percentile) of bilateral ΔbaPWV was 50 (22–102) cm/s. The mean value of baPWV at right side and left side was 1722.3±427.6 and 1695.1±426.9 cm/s respectively (*P*<0.001). The 90 percentile of ΔbaPWV was 185 cm/s. The mean percent errors for right baPWV and left baPWV measurements were 4.0±3.4 and 3.4±3.1% respectively. The differences between patients with and without a ΔbaPWV ≧ 185 cm/s are shown in Table 1. Compared with patients with a ΔbaPWV <185 cm/s, patients with a ΔbaPWV ≧ 185 cm/s were found to have older age, higher prevalence of diabetes mellitus (DM), hypertension and coronary artery disease, higher systolic BP, higher heart rate, higher prevalence of ABI <0.9 (*P*<0.001), higher baPWV (*P*<0.001), higher interankle systolic BP difference (*P*<0.001), higher prevalence of an interankle systolic BP difference ≥15 mmHg (*P*<0.001) [22], higher fasting glucose, lower hematocrit and lower prevalence of eGFR ≧60 mL/min/1.73 m^2^. In addition, patients with a ΔbaPWV ≧ 185 cm/s had higher LAVI (*P*<0.001), higher LVMI (*P*<0.001), higher prevalence of LVH (*P* = 0.004), lower LVEF (*P* = 0.046) and higher E/Ea (*P*<0.001). However, interarm systolic BP difference and the prevalence of an interarm systolic BP difference ≧ 10 mmHg [7] were not significantly different between these two groups.

**Table 1:** Comparison of baseline characteristics between patients with and without a ΔbaPWV ≧ 185 cm/s.

Characteristics	All patients (n = 1111)	Difference <185 cm/s(n = 1000)	Difference ≧ 185 cm/s(n = 111)
ΔbaPWV (cm/s)	50 (22–102)	43 (20–79)	268 (215–432)**
Age (year)	60.7±13.7	59.6±13.3	70.4±13.1**
Male gender (%)	56.6	56.6	56.8
Smoking history (%)	15.1	15.6	10.7
Diabetes mellitus (%)	27.5	25.6	44.1**
Hypertension (%)	68.9	67.5	82.0*
Coronary artery disease (%)	18.5	17.3	29.4*
Systolic BP (mmHg)	135.7±20.9	134.6±20.0	145.6±25.7**
Diastolic BP (mmHg)	77.6±12.0	77.4±11.7	79.0±14.6
Heart rate (beats/min)	69.7±12.4	69.3±12.1	73.3±14.4*
Body mass index (kg/m^2^)	26.0±3.9	26.0±3.9	25.9±4.3
ABI <0.9 (%)	5.0	2.0	32.4**
baPWV (cm/s)	1756.2±448.4	1705.5±405.5	2217.1±544.9**
Interarm systolic BP difference (mmHg)	3 (2–5)	3 (1–5)	3 (2–5)
Interarm systolic BP difference ≥10 mmHg (%)	7.2	7.2	8.1
Interankle systolic BP difference (mmHg)	6 (3–10)	6 (3–10)	10 (5–27)**
Interankle systolic BP difference ≥15 mmHg (%)	13.5	10.9	36.4**
Laboratory parameters			
Fasting glucose (mg/dL)	113.4±40.1	111.4±36.4	132.8±62.9*
Triglyceride (mg/dL)	124 (85.5–184.5)	123 (85–184)	138 (87–186)
Total cholesterol (mg/dL)	192.3±43.8	192.9±43.9	187.4±42.6
Hematocrit (%)	40.4±6.3	40.8±6.3	37.7±5.5**
eGFR ≧60 mL/min/1.73 m^2^ (%)	50.7	53.7	25.0**
Echocardiographic data			
LAVI (ml/m^2^)	35.0±15.2	34.1±14.7	43.1±17.3**
LVMI (g/m^2^)	134.5±45.2	132.3±44.1	154.1±50.6**
LVH (%)	59.4	58.0	72.1*
LVEF (%)	63.1±13.2	63.4±12.8	60.2±16.4*
E/Ea	9.6±5.2	9.3±4.9	12.9±6.7**
E-wave deceleration time (ms)	202.7±63.8	201.6±61.6	212.6±80.6

Abbreviations. baPWV, brachial-ankle pulse wave velocity; blood pressure; ABI, ankle-brachial index; eGFR, estimated glomerular filtration rate; LAVI, left atrial volume index; LVMI, left ventricular mass index; LVH, left ventricular hypertrophy; LVEF, left ventricular ejection fraction; E, transmitral E wave velocity; Ea, early diastolic mitral annulus velocity.

*P<0.05, **P<0.001 compared patients with a bilateral baPWV difference <185 cm/s.

Table 2 displays the odds ratio (OR) estimates of ABI <0.9, baPWV, LVMI and interankle systolic BP difference ≧ 15 mmHg for a ΔbaPWV ≧ 185 cm/s in all study patients with adjustment for demographic, clinical and biochemical parameters. We performed three multivariate analyses. In the first multivariate analysis (covariates included age, sex, diabetes, hypertension, coronary artery disease, systolic blood pressure, heart rate, ABI <0.9, baPWV, fasting glucose, hematocrit and eGFR ≧60 mL/min/1.73 m^2^), the ABI <0.9 (OR, 33.001; *P*<0.001) and baPWV (per 10 cm/s; OR, 1.019; *P*<0.001) were associated with a ΔbaPWV ≧ 185 cm/s. In the second multivariate analysis (covariates included age, sex, diabetes, hypertension, coronary artery disease, systolic blood pressure, heart rate, fasting glucose, hematocrit, eGFR ≧60 mL/min/1.73 m^2^ and LVMI), LVMI was associated with a ΔbaPWV ≧ 185 cm/s (OR, 1.006; *P* = 0.021). Besides, in the third multivariate analysis (covariates included age, sex, diabetes, hypertension, coronary artery disease, systolic blood pressure, heart rate, interankle systolic BP difference ≧ 15 mmHg, fasting glucose, hematocrit and eGFR ≧60 mL/min/1.73 m^2^), interankle systolic BP difference ≧ 15 mmHg was associated with a ΔbaPWV ≧ 185 cm/s (OR, 2.016; *P* = 0.026).

**Table 2:** Determinants of ΔbaPWV ≧ 185 cm/s in all study patients.

Parameter	Multivariate: Model 1		Multivariate: Model 2		Multivariate : Model 3	
	OR (95% CI)	*P*	OR (95% CI)	*P*	OR (95% CI)	*P*
Age (per 1 year)	0.998 (0.968–1.029)	0.898	1.054 (1.029–1.079)	<0.001	1.044 (1.019–1.070)	<0.001
Male gender	0.932 (0.500–1.738)	0.825	0.862 (0.497–1.493)	0.596	0.969 (0.556–1.687)	0.911
Diabetes mellitus	0.991 (0.480–2.048)	0.981	1.132 (0.606–2.117)	0.697	1.284 (0.682–2.418)	0.439
Hypertension	1.666 (0.730–3.804)	0.225	1.755 (0.847–3.636)	0.130	1.662 (0.781–3.536)	0.188
Coronary artery disease	1.891 (0.906–3.948)	0.090	2.234 (1.189–4.199)	0.013	2.263 (1.191–4.297)	0.013
Systolic blood pressure (per 1 mmHg)	1.001 (0.984–1.019)	0.898	1.021 (1.008–1.035)	0.002	1.023 (1.009–1.036)	0.001
Heart rate (per beat/min)	1.006 (0.979–1.033)	0.682	1.027 (1.005–1.049)	0.016	1.025 (1.002–1.048)	0.034
ABI <0.9	33.001 (12.879–84.605)	<0.001	–	–	–	–
baPWV (per 10 cm/s)	1.019 (1.011–1.028)	<0.001	–	–	–	–
Interankle systolic BP difference ≥15 mmHg	–	–	–	–	2.016 (1.085–3.744)	0.026
Laboratory parameters						
Fasting glucose (mg/dL)	1.006 (1.000–1.013)	0.052	1.009 (1.003–1.015)	0.002	1.005 (0.999–1.011)	0.088
Hematocrit (per 1%)	0.982 (0.923–1.046)	0.577	0.970 (0.920–1.023)	0.260	0.966 (0.915–1.020)	0.211
eGFR ≧60 mL/min/1.73 m^2^	0.906 (0.459–1.787)	0.775	0.819 (0.449–1.496)	0.517	0.704 (0.374–1.323)	0.275
Echocardiographic data						
LVMI (per 1 g/m^2^)	–	–	1.006 (1.001–1.011)	0.021	–	–

Values expressed as odds ratio (OR) and 95% confidence interval (CI). Abbreviations are the same as in Table 1.

Covariates in the basic model included age, sex, diabetes, hypertension, coronary artery disease, systolic blood pressure, heart rate, fasting glucose, hematocrit and eGFR ≧60 mL/min/1.73 m^2^.

Covariates in the model 1 included covariates in the basic model+ABI <0.9 and baPWV.

Covariates in the model 2 included covariates in the basic model+LVMI.

Covariates in the model 3 included covariates in the basic model+interankle systolic BP difference ≥15 mmHg.

In addition, we performed analyses using 85 percentile (ΔbaPWV ≧ 140 cm/s) or 95 percentile (ΔbaPWV ≧ 269 cm/s) as a cut-off value of bilateral baPWV difference. Multivariate analysis showed the association of ABI <0.9 (*P*<0.001) and high baPWV (*P*<0.001) with bilateral baPWV difference ≧ 85 percentile still existed, but the association of LVMI (*P* = 0.493) and interankle systolic BP difference ≧ 15 mmHg (*P* = 0.125) did not reach statistical significance. Besides, the association of ABI <0.9 (*P*<0.001), high baPWV (*P*<0.001) and interankle systolic BP difference ≧ 15 mmHg (*P*<0.001) with bilateral baPWV difference ≧ 95 percentile still existed, but the association of LVMI (*P* = 0.274) did not reach statistical significance.

Since a low ABI might diminish baPWV accuracy, [23] we further performed a subgroup analysis after excluding 56 patients with ABI <0.9. We found LVMI was still independently associated with a ΔbaPWV ≧ 185 cm/s after adjusting for demographic, clinical and biochemical factors (OR, 1.007; *P* = 0.019).

## Discussion

In the present study, using a simultaneous measurement technique, we found that ABI <0.9, high baPWV, high LVMI and an interankle systolic BP difference ≥15 mmHg were independently associated with a large bilateral baPWV difference, but an interarm systolic BP difference ≥10 mmHg was not. The relation between ΔbaPWV ≧ 185 cm/s and high LVMI still existed even after excluding patients with ABI <0.9.

Arterial stiffness measured by pulse wave velocity (PWV) in different segments appeared to be differentially associated with micro- and macro-vascular complications [24]. Pannier et al. revealed that aortic PWV, but not femorotibial PWV, was an independent predictor for cardiovascular death in hemodialysis patients [6]. Kim et al. had also shown that diabetic retinopathy was closely associated with heart-femoral PWV, but not carotid-brachial PWV [25]. Besides, Hatsuda et al. found the PWVs of different segments correlated with each other in patients without ischemic heart disease, but less impressive in those with ischemic heart disease [4]. This finding suggested that unequal arterial stiffness might be related to ischemic heart disease. One important finding of our study was that there was a significant association of ABI <0.9 and high baPWV with a large bilateral baPWV difference. The reason for the association of ABI <0.9 and larger bilateral baPWV difference may be related to a lower baPWV value at the side of ABI <0.9 [23], which contributed to larger bilateral baPWV difference. The ABI <0.9 and baPWV are good markers of peripheral artery occlusive disease and arterial stiffness respectively [2], [15], [26]–[28]. A lower ABI and high baPWV show strong powers in predicting the mortality in various populations [29]–[32]. Hence, unequal arterial stiffness indicated by a large bilateral baPWV difference may be associated with peripheral vascular disease and have a potential to become a useful parameter in predicting adverse cardiovascular events.

Arterial stiffness may contribute to LVH independently of BP [33], [34]. Some studies have reported a positive correlation between PWV and increased LVMI and LVH [35]–[37]. Arterial stiffness is associated with hypertrophy and atherosclerosis within the capacitance arteries that result in an increase in PWV and consequent alterations in the pressure waveform and increases in systolic and pulse pressure. Alterations in wave reflections combined with increased stiffness may also contribute to LVH [33], [38], [39]. Previous studies using different measurement methods of arterial stiffness have shown increased arterial stiffness was independently associated with LVH in a variety of populations [35], [36], [40]–[42]. Our study showed that a large bilateral baPWV difference was associated with LVMI. The relationship between a large bilateral baPWV difference and LVMI still existed even after excluding patients with ABI <0.9. This is the first study to show the association between unequal arterial stiffness with LVMI. Hence, LVH might represent a causal intermediary between unequal arterial stiffness and poor cardiovascular outcomes.

Several studies have reported that an interarm BP difference is associated with subclavian stenosis diagnosed by angiography, peripheral artery disease by ABI and LVH by echocardiography [11], [43]. Furthermore, an interarm BP difference is strongly associated with increased cardiovascular mortality and all-cause mortality [43]. Besides, Sheng et al. have recently found an interankle systolic BP difference predicts mortality in the elderly [12]. Our recent study has also reported that a difference in systolic BP ≧ 15 mmHg or diastolic BP of ≧ 10 mmHg between ankles is associated with atherosclerosis and increased risk for overall and cardiovascular mortality in hemodialysis patients [13]. Unequal limb atherosclerosis may represent a causal intermediary between an interarm or interankle systolic BP difference and poor cardiovascular outcomes, but it has never been validated. Our present study showed that a large interankle, but not interarm, systolic BP difference was associated with a large bilateral baPWV difference. It might imply only patients with an interankle systolic BP difference ≧ 15 mmHg had a significantly unequal arterial stiffness indicated by large ΔbaPWV. Hence, the association between interankle systolic BP difference and poor cardiovascular outcomes may be explained by unequal arterial stiffness. However, the same mechanism may not be able to explain the association between interarm systolic BP difference and poor cardiovascular outcomes. In addition, there was a lot more overlap of artery territory in calculating ΔbaPWV and interankle systolic BP difference than in calculating ΔbaPWV and interarm systolic BP difference, which might explain why a large interankle, but not interarm, systolic BP difference was associated with a large ΔbaPWV in the present study. However, the influence of the asymmetrical aortic arch geometry on the bilateral baPWV difference could not be excluded, even though an interarm systolic BP difference was not related to bilateral baPWV difference.

There were several limitations to this study. First, this study was cross-sectional, so the causal relationship and long-term clinical outcomes could not be confirmed. Future prospective studies are needed to address the issues. Second, as no studies have documented the reliable abnormal values of a bilateral baPWV difference, we used 90 percentile of bilateral baPWV difference to classify our study patients. Third, we had no data of direct comparison between bilateral baPWV and carotid-femoral PWV difference. Fourth, the travelled path was longer at the right than at the left body side [44], which might contribute to the bilateral baPWV difference. Finally, since the subjects of this study were already being evaluated for heart disease by echocardiography, it was susceptible to selection bias, making findings potentially less generalized.

In conclusion, our results demonstrated that ABI <0.9, high baPWV and high LVMI were significantly correlated with unequal arterial stiffness. In addition, interankle, but not interarm, systolic BP difference was associated with unequal arterial stiffness.

## References

[1] Shoji T, Emoto M, Shinohara K, Kakiya R, Tsujimoto Y, et al (2001) Diabetes mellitus, aortic stiffness, and cardiovascular mortality in end-stage renal disease. J Am Soc Nephrol 12: 2117–2124. 11562410 10.1681/ASN.V12102117

[2] Boutouyrie P, Tropeano AI, Asmar R, Gautier I, Benetos A, et al (2002) Aortic stiffness is an independent predictor of primary coronary events in hypertensive patients: a longitudinal study. Hypertension 39: 10–15. 11799071 10.1161/hy0102.099031

[3] Cruickshank K, Riste L, Anderson SG, Wright JS, Dunn G, et al (2002) Aortic pulse-wave velocity and its relationship to mortality in diabetes and glucose intolerance: an integrated index of vascular function? Circulation 106: 2085–2090. 12379578 10.1161/01.cir.0000033824.02722.f7

[4] Hatsuda S, Shoji T, Shinohara K, Kimoto E, Mori K, et al (2006) Regional arterial stiffness associated with ischemic heart disease in type 2 diabetes mellitus. J Atheroscler Thromb 13: 114–121. 16733300 10.5551/jat.13.114

[5] Kingwell BA, Waddell TK, Medley TL, Cameron JD, Dart AM (2002) Large artery stiffness predicts ischemic threshold in patients with coronary artery disease. J Am Coll Cardiol 40: 773–779. 12204510 10.1016/s0735-1097(02)02009-0

[6] Pannier B, Guerin AP, Marchais SJ, Safar ME, London GM (2005) Stiffness of capacitive and conduit arteries: prognostic significance for end-stage renal disease patients. Hypertension 45: 592–596. 15753232 10.1161/01.HYP.0000159190.71253.c3

[7] Clark CE, Campbell JL, Evans PH, Millward A (2006) Prevalence and clinical implications of the inter-arm blood pressure difference: A systematic review. J Hum Hypertens 20: 923–931. 17036043 10.1038/sj.jhh.1002093

[8] Clark CE, Campbell JL, Powell RJ (2007) The interarm blood pressure difference as predictor of cardiovascular events in patients with hypertension in primary care: cohort study. J Hum Hypertens 21: 633–638. 17460712 10.1038/sj.jhh.1002209

[9] Clark CE, Greaves CJ, Evans PH, Dickens A, Campbell JL (2009) Inter-arm blood pressure difference in type 2 diabetes: a barrier to effective management? Br J Gen Pract 59: 428–432. 19520026 10.3399/bjgp09X420752 PMC2688045

[10] Aboyans V, Criqui MH, McDermott MM, Allison MA, Denenberg JO, et al (2007) The vital prognosis of subclavian stenosis. J Am Coll Cardiol 49: 1540–1545. 17418292 10.1016/j.jacc.2006.09.055

[11] Su HM, Lin TH, Hsu PC, Chu CY, Lee WH, et al (2012) Association of interarm systolic blood pressure difference with atherosclerosis and left ventricular hypertrophy. PloS one 7: e41173. 22927905 10.1371/journal.pone.0041173 PMC3426512

[12] Sheng CS, Liu M, Zeng WF, Huang QF, Li Y, et al (2013) Four-Limb Blood Pressure as Predictors of Mortality in Elderly Chinese. Hypertension 61: 1155–1160. 23569084 10.1161/HYPERTENSIONAHA.111.00969

[13] Chen SC, Chang JM, Tsai YC, Tsai JC, Su HM, et al (2012) Association of interleg BP difference with overall and cardiovascular mortality in hemodialysis. Clin J Am Soc Nephrol 7: 1646–1653. 22859748 10.2215/CJN.04570512 PMC3463210

[14] Yamashina A, Tomiyama H, Takeda K, Tsuda H, Arai T, et al (2002) Validity, reproducibility, and clinical significance of noninvasive brachial-ankle pulse wave velocity measurement. Hypertens Res 25: 359–364. 12135313 10.1291/hypres.25.359

[15] Fishbane S, Youn S, Kowalski EJ, Frei GL (1995) Ankle-arm blood pressure index as a marker for atherosclerotic vascular diseases in hemodialysis patients. Am J Kidney Dis 25: 34–39. 7810530 10.1016/0272-6386(95)90622-3

[16] Devereux RB, Alonso DR, Lutas EM, Gottlieb GJ, Campo E, et al (1986) Echocardiographic assessment of left ventricular hypertrophy: comparison to necropsy findings. Am J Cardiol 57: 450–458. 2936235 10.1016/0002-9149(86)90771-x

[17] Mancia G, De Backer G, Dominiczak A, Cifkova R, Fagard R, et al (2007) 2007 Guidelines for the Management of Arterial Hypertension: The Task Force for the Management of Arterial Hypertension of the European Society of Hypertension (ESH) and of the European Society of Cardiology (ESC). J Hypertens 25: 1105–1187. 17563527 10.1097/HJH.0b013e3281fc975a

[18] Lang RM, Bierig M, Devereux RB, Flachskampf FA, Foster E, et al (2005) Recommendations for chamber quantification: a report from the American Society of Echocardiography’s Guidelines and Standards Committee and the Chamber Quantification Writing Group, developed in conjunction with the European Association of Echocardiography, a branch of the European Society of Cardiology. J Am Soc Echocardiogr 18: 1440–1463. 16376782 10.1016/j.echo.2005.10.005

[19] Verberk WJ, Kessels AG, Thien T (2011) Blood pressure measurement method and inter-arm differences: a meta-analysis. Am J Hypertens 24: 1201–1208. 21776035 10.1038/ajh.2011.125

[20] Vickery S, Stevens PE, Dalton RN, van Lente F, Lamb EJ (2006) Does the ID-MS traceable MDRD equation work and is it suitable for use with compensated Jaffe and enzymatic creatinine assays? Nephrol Dial Transplant 21: 2439–2445. 16720592 10.1093/ndt/gfl249

[21] Levey AS, Bosch JP, Lewis JB, Greene T, Rogers N, et al (1999) A more accurate method to estimate glomerular filtration rate from serum creatinine: a new prediction equation. Modification of Diet in Renal Disease Study Group. Ann Intern Med 130: 461–470. 10075613 10.7326/0003-4819-130-6-199903160-00002

[22] Zhang Z, Ma J, Tao X, Zhou Y, Liu X, et al (2013) The prevalence and influence factors of interankle systolic blood pressure difference in community population. PLoS One 8: e70777. 24015177 10.1371/journal.pone.0070777 PMC3754990

[23] Motobe K, Tomiyama H, Koji Y, Yambe M, Gulinisa Z, et al (2005) Cut-off value of the ankle-brachial pressure index at which the accuracy of brachial-ankle pulse wave velocity measurement is diminished. Circ J 69: 55–60. 15635203 10.1253/circj.69.55

[24] Tsuchikura S, Shoji T, Kimoto E, Shinohara K, Hatsuda S, et al (2010) Central versus peripheral arterial stiffness in association with coronary, cerebral and peripheral arterial disease. Atherosclerosis 211: 480–485. 20430390 10.1016/j.atherosclerosis.2010.03.037

[25] Kim WJ, Park CY, Park SE, Rhee EJ, Lee WY, et al (2012) The association between regional arterial stiffness and diabetic retinopathy in type 2 diabetes. Atherosclerosis 225: 237–241. 23017354 10.1016/j.atherosclerosis.2012.08.034

[26] Fowkes FG, Housley E, Cawood EH, Macintyre CC, Ruckley CV, et al (1991) Edinburgh Artery Study: prevalence of asymptomatic and symptomatic peripheral arterial disease in the general population. Int J Epidemiol 20: 384–392. 1917239 10.1093/ije/20.2.384

[27] Newman AB, Tyrrell KS, Kuller LH (1997) Mortality over four years in SHEP participants with a low ankle-arm index. J Am Geriatr Soc 45: 1472–1478. 9400557 10.1111/j.1532-5415.1997.tb03198.x

[28] Lehmann ED (1999) Clinical value of aortic pulse-wave velocity measurement. Lancet 354: 528–529. 10470691 10.1016/S0140-6736(99)00179-8

[29] Bosevski M, Peovska I (2013) Clinical Usefulness of Assessment of Ankle-Brachial Index and Carotid Stenosis in Type 2 Diabetic Population-Three-Year Cohort Follow-Up of Mortality. Angiology 64: 64–68. 22323833 10.1177/0003319711435936

[30] Kitahara T, Ono K, Tsuchida A, Kawai H, Shinohara M, et al (2005) Impact of brachial-ankle pulse wave velocity and ankle-brachial blood pressure index on mortality in hemodialysis patients. Am J Kidney Dis 46: 688–696. 16183424 10.1053/j.ajkd.2005.06.016

[31] Pasqualini L, Schillaci G, Pirro M, Vaudo G, Leli C, et al (2012) Prognostic value of low and high ankle-brachial index in hospitalized medical patients. Eur J Intern Med 23: 240–244. 22385881 10.1016/j.ejim.2011.09.004

[32] Chen SC, Chang JM, Hwang SJ, Tsai JC, Liu WC, et al (2010) Ankle brachial index as a predictor for mortality in patients with chronic kidney disease and undergoing haemodialysis. Nephrology (Carlton) 15: 294–299. 20470297 10.1111/j.1440-1797.2010.01187.x

[33] Roman MJ, Ganau A, Saba PS, Pini R, Pickering TG, et al (2000) Impact of arterial stiffening on left ventricular structure. Hypertension 36: 489–494. 11040224 10.1161/01.hyp.36.4.489

[34] Roman MJ, Pickering TG, Schwartz JE, Pini R, Devereux RB (1995) Association of carotid atherosclerosis and left ventricular hypertrophy. J Am Coll Cardiol 25: 83–90. 7798531 10.1016/0735-1097(94)00316-i

[35] Chung CM, Lin YS, Chu CM, Chang ST, Cheng HW, et al (2012) Arterial Stiffness Is the Independent Factor of Left Ventricular Hypertrophy Determined by Electrocardiogram. Am J Med Sci 344: 190–193. 22270392 10.1097/MAJ.0b013e318242a354

[36] Dellegrottaglie S, Sands RL, Gillespie BW, Gnanasekaran G, Zannad F, et al (2011) Association between markers of collagen turnover, arterial stiffness and left ventricular hypertrophy in chronic kidney disease (CKD): the Renal Research Institute (RRI)-CKD study. Nephrol Dial Transplant 26: 2891–2898. 21562144 10.1093/ndt/gfr186

[37] Masugata H, Senda S, Hoshikawa J, Murao K, Hosomi N, et al (2010) Elevated brachial-ankle pulse wave velocity is associated with left ventricular hypertrophy in hypertensive patients after stroke. Tohoku J Exp Med 220: 177–182. 20208411 10.1620/tjem.220.177

[38] Avolio AP, Chen SG, Wang RP, Zhang CL, Li MF, et al (1983) Effects of aging on changing arterial compliance and left ventricular load in a northern Chinese urban community. Circulation 68: 50–58. 6851054 10.1161/01.cir.68.1.50

[39] Kelly R, Hayward C, Avolio A, O’Rourke M (1989(Noninvasive determination of age-related changes in the human arterial pulse. Circulation 80: 1652–1659. 2598428 10.1161/01.cir.80.6.1652

[40] Matsui Y, Ishikawa J, Shibasaki S, Shimada K, Kario K (2011) Association between home arterial stiffness index and target organ damage in hypertension: comparison with pulse wave velocity and augmentation index. Atherosclerosis 219: 637–642. 21978919 10.1016/j.atherosclerosis.2011.09.027

[41] Su HM, Lin TH, Hsu PC, Lee CS, Lee WH, et al.. (2013) Association of Brachial-Ankle Pulse Wave Velocity, Ankle-Brachial Index and Ratio of Brachial Pre-Ejection Period to Ejection Time With Left Ventricular Hypertrophy. Am J Med Sci [Epub of ahead]. 10.1097/MAJ.0b013e31828c5bee 23588262

[42] Su HM, Lin TH, Hsu PC, Chu CY, Lee WH, et al (2012) Impact of systolic time intervals on the relationship between arterial stiffness and left ventricular hypertrophy. Atherosclerosis 223: 171–176. 22633473 10.1016/j.atherosclerosis.2012.04.022

[43] Clark CE, Taylor RS, Shore AC, Ukoumunne OC, Campbell JL (2012) Association of a difference in systolic blood pressure between arms with vascular disease and mortality: a systematic review and meta-analysis. Lancet 379: 905–914. 22293369 10.1016/S0140-6736(11)61710-8

[44] Bossuyt J, Van De Velde S, Azermai M, Vermeersch SJ, De Backer TL, et al (2013) Noninvasive assessment of carotid-femoral pulse wave velocity: the influence of body side and body contours. J Hypertens 31: 946–951. 23511340 10.1097/HJH.0b013e328360275d

